# In situ, high-resolution evidence for iron-coupled mobilization of phosphorus in sediments

**DOI:** 10.1038/srep24341

**Published:** 2016-04-18

**Authors:** Shiming Ding, Yan Wang, Dan Wang, Yang Yang Li, Mengdan Gong, Chaosheng Zhang

**Affiliations:** 1 State Key Laboratory of Lake Science and Environment, Nanjing Institute of Geography and Limnology, Chinese Academy of Sciences, Nanjing 210008, China; 2 School of Chemical Engineering, Nanjing University of Science and Technilogy, Nanjing 210094, China; 3 GIS Centre, Ryan Institute and School of Geography and Archaeology, National University of Ireland, Galway, Ireland

## Abstract

Reductive dissolution of phosphorus-bearing iron (Fe) (oxyhydr)oxides has been regarded as a primary mechanism responsible for the mobilization of phosphorus (P) in sediments for over 70 years. However, to date there is little *in situ* evidence to support this hypothesis. In this study, a total of 16 sites in the large eutrophic Lake Taihu were selected for investigation. Newly-developed diffusive gradients in thin films (ZrO-Chelex DGT) probes were deployed to simultaneously measure labile Fe and P mainly released from sediment solids at millimeter spatial resolution. Significantly positive correlations were observed between DGT-labile Fe and P at 14 sites, implying a release of P following reductive dissolution of Fe (oxyhydr)oxides. A coincident resupply of Fe(II) and P was observed from sediment solids to buffer their releases from DGT perturbance, further verifying the mechanism of Fe-coupled mobilization of P. The ratio of DGT-labile Fe/P was found to be positively correlated with the ratio of easily reducible (oxyhydr)oxide Fe to its associated P, indicating that this solid phase should retain P prior to its release. The results provide direct evidence for the coupling between Fe and P in sediments and further identify the easily reducible Fe (oxyhydr)oxide species involved in the coupling process.

Eutrophication is simply defined by the enrichment of natural waters with plant nutrients (phosphorus and nitrogen) from natural processes and anthropogenic activities^1^. It is one of the most widespread environmental problems of inland waters and urgently needs to be solved, especially in developing countries with respect to water resource protection^2^,^3^. Phosphorus (P) plays a critical role in limiting primary productivity and regulating the trophic status of lakes^4^,^5^. The occurrence of eutrophication was attributed mainly to point and nonpoint discharges of P-rich wastewater before the 1990s. Measures to solve the eutrophication problem have focused on reducing the external sources, whereas a delay in recovery has been found for many lakes after the reduction of external P loading. It is recognized that P release from sediments is the major process responsible for this delay. This process persists for at least 5–10 years, and typically 10–15 years following the external P load reduction according to studies of lakes in Europe and North America^6^,^7^. P release from sediments can contribute the majority (up to 80%) of the total P input in some lakes, and becomes the main driver of primary production, especially when the bottom waters become anoxic in the summer^8^,^9^.

The mechanisms behind the release of P have been explored for over 70 years. Einsele^10^ first proposed the coupling between iron (Fe) and P cycles that is responsible for the retention and mobilization of P in sediments. Mortimer^11^ refined this concept, in which the retention of P was attributed to strong adsorption by iron (oxyhy)droxides in surface sediments under oxic conditions, whereas the reduction of iron (oxyhy)droxides under anoxic conditions led to the release of both P and Fe(II) into the pore water and an upward diffusion to the water column. Petticrew and Arocena^12^ later provided evidence for the relationships of P release from sediments with hypolimnetic anoxia and Fe-bound P. Rydin^13^ identified Fe-bound P as one of the major potentially-mobile forms of P in lake sediments. Spears *et al*.^14^ observed that Fe-bound P is a temporal P pool responsible for the short-term, high-magnitude P release occurring in late summer and winter under anoxic sediment conditions. The coupling of P mobility with Fe cycling was further supported by the coincident behaviors between them in surface sediments and bottom waters, such as 1) strong correlations between reactive P and reactive Fe in surface sediments^15^ and between hypolimnetic P and Fe^16^, 2) similar distributions of soluble Fe and P in pore waters^17^, and 3) simultaneous release of P and Fe from sediments under anoxic conditions^15^,^18^. However, several recent studies suggested that the Fe-coupled P mobilization in sediments simultaneously relies on the conversion of Fe compounds into Fe-sulfides via bacterial sulfate reduction^19^, the sorption of P by aluminum hydroxides under anoxic conditions^20^, and the presence of nitrate in the hypolimnion^21^. The release of P is also considered to be more complex than the concept of Fe-P coupling^22^. This process involves alternative release mechanisms such as dissolution of calcium-bound P^23^, release of bacterial P^24^, and decomposition of organic P^25^. In this case, a further examination of the existence of Fe-P coupling is particularly required.

Interpretations of the relationship between Fe and P in sediments are mostly based on geochemical modeling^12^, chemical fractionation^13^,^15^,^18^,^26^,^27^ and adsorption experiments^28^ in combination with field investigation, intact core incubation or sediment sample tests. A few studies employed dialysis (Peeper) and *in situ* observations of the co-distribution between soluble Fe and P in pore water^17^, but the Peeper measurement cannot reflect the solid sources resupplying the pore water with P. Until now, there has been a lack of *in situ* evidence to support the mechanism of Fe-coupled mobilization of P in sediments. Diffusive gradients in thin films (DGT) is a dynamic sampling technique capable of *in situ* sampling of P, metals and metalloids in sediments at a high spatial resolution^29^,^30^. DGT can pre-concentrate analytes and reduce the problems associated with conventional sampling methods during the sampling process, such as analyte contamination and speciation change^31^. Furthermore, a new DGT (ZrO-Chelex DGT) has been developed for simultaneous measurements of Fe and P in sediments at the same time and position^32^, which offers a substantial advantage in investigations of their relationships.

The aim of this study was to provide *in situ* evidence for the coupling of Fe to P mobilization in sediments based on the use of ZrO-Chelex DGT^32^. *In situ* deployment of the DGT probes was performed in eutrophic Lake Taihu to simultaneously obtain the distributions of labile Fe and P mainly released from sediment solids at a millimeter scale. Dynamics of Fe and P release from sediment solids were examined. The Fe (oxyhydr)oxide species retaining the released P were further identified. The results finally led to the determination of the mechanism of Fe-coupled mobilization of P in sediments.

## Results and Discussion

### Sediment properties

The chemical properties of the sediments at each site are listed in Table 1. The pH values ranged from 6.59 to 7.46. The sediment TOC content varied from 4.59 to 23.37 mg g^−1^, with the three greatest values at two sites (12 and 16) dominated by submerged macrophytes and one site (3) dominated by algal blooms. The pseudo total Ca, Al and Mn contents ranged from 4.3 to 16.09 mg g^−1^, 49.46 to 70.27 mg g^−1^ and 0.37 to 1.28 mg g^−1^, respectively. The ranges of these properties are similar to those reported previously for Lake Taihu^33^.

### Sediment Fe and P

The information on sediment Fe and P is summarized in Table 2. The total content of Fe in sediments for the 16 sites ranged from 25.14 to 45.44 mg g^−1^, with an average of 30.68 mg g^−1^. The range is similar to the 20.7 to 42.7 mg g^−1^ reported by Bai *et al*.^33^ in Lake Taihu. The content of total P ranged from 0.36 to 0.95 mg g^−1^. The greatest value was found at Site 3 in the northern bay. High values of 14.73 and 15.52 mg g^−1^ have been reported in the sediments of this bay^33^,^34^, reflecting the heavily polluted character of these sediments in comparison with the other sites. The ratio of total Fe to total P concentration in the sediments ranged from 32.1 to 92.7, with the lowest values at Site 3. The ratios are much greater than the critical value of 15 suggested by Jensen *et al*.^35^ for determining whether the oxic surface sediments had sufficient adsorption ability to control the release of P to the overlying waters.

The concentration distributions of easily reducible (Fe-ox1) and reducible Fe (oxyhydr)oxides (Fe-ox2) and their associated P (P-ox1, P-ox2) in sediments with depth are shown in Supplementary Figs S1 and S2. Their average values of each site are listed in Table 2. The two types of Fe (oxyhydr)oxides capable of retention of P are mainly 1) ferrihydrite, and 2) goethite and hematite^36^. Both the Fe-ox1 and P-ox1exhibited decreasing trends with the increase of depth at Sites 2, 4 and 6 throughout the profiles and at Sites 3 and 12 in parts of the profiles, whereas other sites showed a small fluctuation or irregularly changing trend (Supplementary Fig. S1). The average concentrations of Fe-ox1 and P-ox1 for all of the sites were 5.77 mg g^−1^ and 0.25 mg g^−1^, accounting for 18.7% and 46.3% of the total contents, respectively (Table 2). Their concentration ratios ranged from 11.7 to 43.2, which are much lower than the ratios of total Fe and P in sediments. More irregular fluctuations with depth were found for the Fe-ox2 and P-ox2, and only Site 4 showed an overall decreasing trend (Supplementary Fig. S2). The average values of Fe-ox2 and P-ox2 in all the sites were 6.78 mg g^−1^ (Fe) and 0.08 mg g^−1^ (P), accounting for respectively 22.7% and 15.5% of their total contents in sediments (Table 2). Their concentration ratios ranged from 63.6 to 129.0, which are much greater than the ratios of total Fe and P in sediments.

Both easily reducible or reducible Fe and their associated P exhibited similar patterns of changes with depth at most sites (Supplementary Figs S1 and S2). Accordingly, significantly positive correlations were found between them across the whole depths or from the SWI to a middle depth range in these sites (Supplementary Figs S3 and S4). This may reflect that the partitioning of P in sediments was regulated by the two types of Fe (oxyhydr)oxides. It should be noted that a positive correlation existed between total P and P-ox1 (R^2^ = 0.80, *p* < 0.001). Moreover, there was a negative correlation between the proportions of P-ox1 and P-ox2 (R^2^ = 0.39, *p* < 0.05). In addition, there is more P associated with Fe-ox1 than with Fe-ox2, despite the similar concentrations of the two Fe (oxyhydr)oxide fractions. These results demonstrate that the Fe-ox1 may preferentially bind P and play a primary role in the partitioning of the P pool in sediments of Lake Taihu. This may be because ferrihydrite has a greater capacity and higher specific adsorption capacity for phosphate in comparison with goethite and hematite due to a poor crystallinity, large micropore volume and large surface site density^37^.

### Distribution of DGT-labile Fe and P

**Figure 1:**
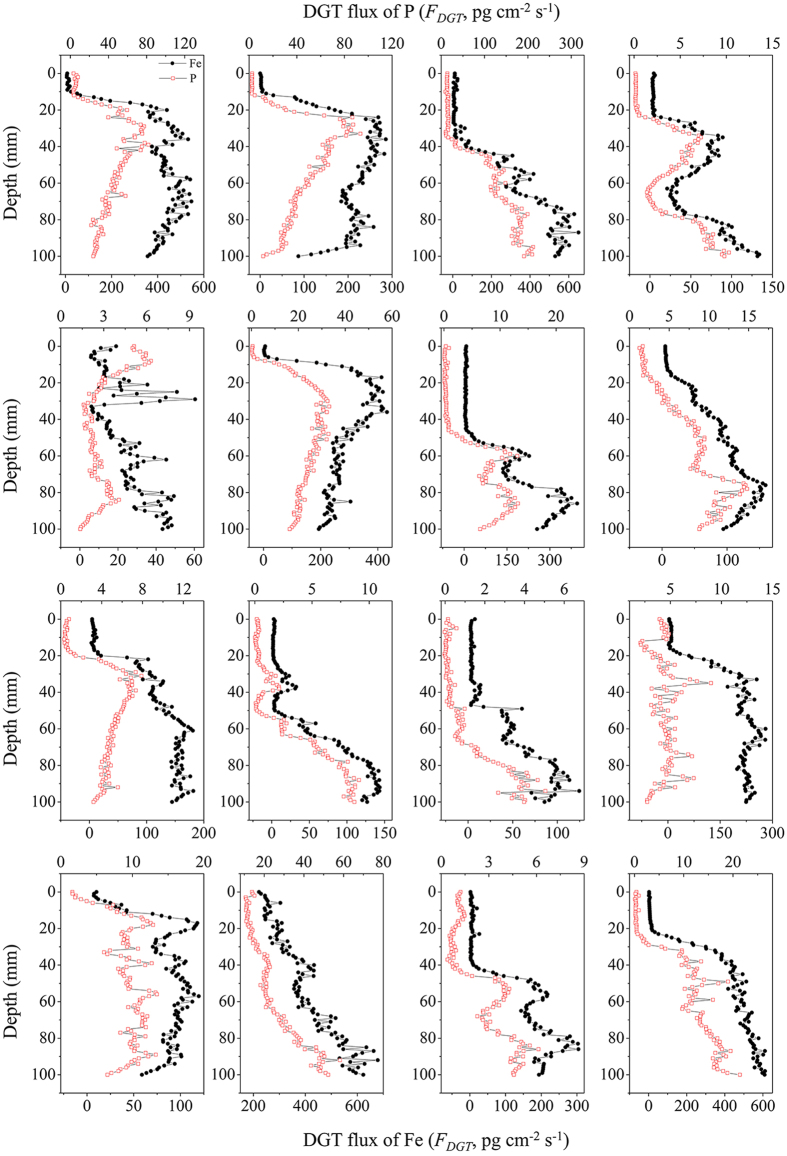
One-dimensional distributions of DGT-labile Fe and P in sediments of Lake Taihu.

The 1D distributions of DGT-labile Fe and P (interpreted as *F*_DGT_) collected from the 16 sampling sites in Lake Taihu are shown in Fig. 1. For all of the sites, the *F*_DGT_ values of labile Fe are much greater than those of labile P, but their difference is variable across the sites investigated. The highest *F*_DGT_ to the DGT probes were observed in the bottom of Site 3, approaching 0.68 ng cm^−2^ s^−1^ for Fe and 0.21 ng cm^−2^ s^−1^ for P; the capacities of the ZrO-Chelex DGT to take up the two types of solutes was not exceeded at any point in this study. The distributions of both Fe and P exhibited appreciable variation with depth at each site. There was an increasing trend with depth down to the sediment bottom at Sites 3, 7, 8, 10, 11, 14, 15 and 16 for both Fe and P, and at Site 5 for Fe. There was also an increasing trend from the SWI to middle depths varying from 20 mm to 60 mm, followed by a stable stage or a decrease down to the sediment bottom at Sites 1, 2, 6 and 9 for both Fe and P and at Site 12 for Fe. Other P or Fe profiles exhibited irregular distribution patterns (Sites 4 and 5) or remained stable with depth (Site 12).

**Figure 2:**
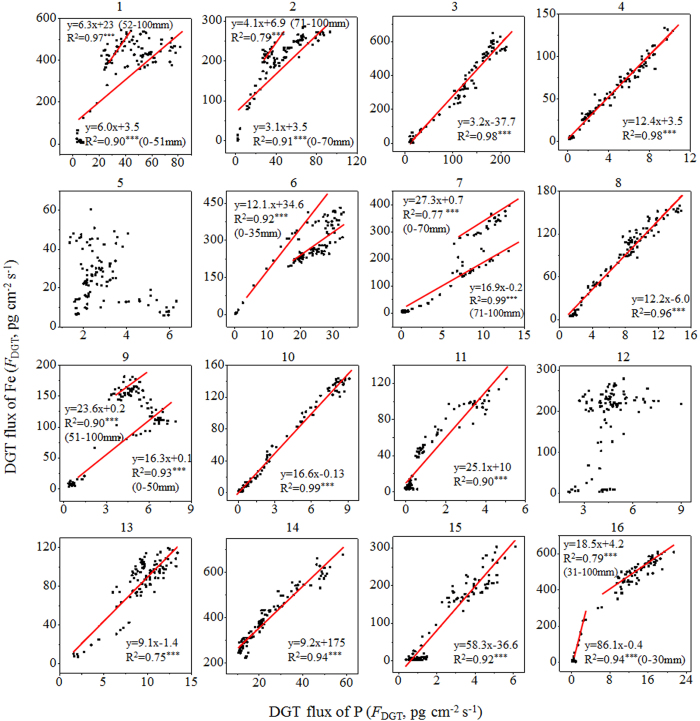
Correlation analysis between DGT-labile Fe and P.

Strong variations in the vertical distributions of labile Fe or P were observed (Fig. 1). Despite this, there was a coincident distribution between them at all of the sites except for 5 and 12. Such a feature was highlighted by the corresponding changes at their localized maximum or minimum concentration positions. For example, the *F*_DGT_ of both Fe and P had peaks at 33 mm depth at Site 4, at 60 mm and 87 mm at Site 7, at 79 mm at Site 8, and at 54 mm and 85 mm at Site 15. Their positive relationships can be fitted using a line for 9 sites (3, 4, 8 and 10–15) or using two lines for 6 sites (1, 2, 6, 7, 9 and 16), all with significance levels of *p* < 0.001 (Fig. 2). The slopes in the linear equations had a range from 3.0 to 86.1, which also reflected the values of the Fe/P ratio from DGT measurement.

**Figure 3:**
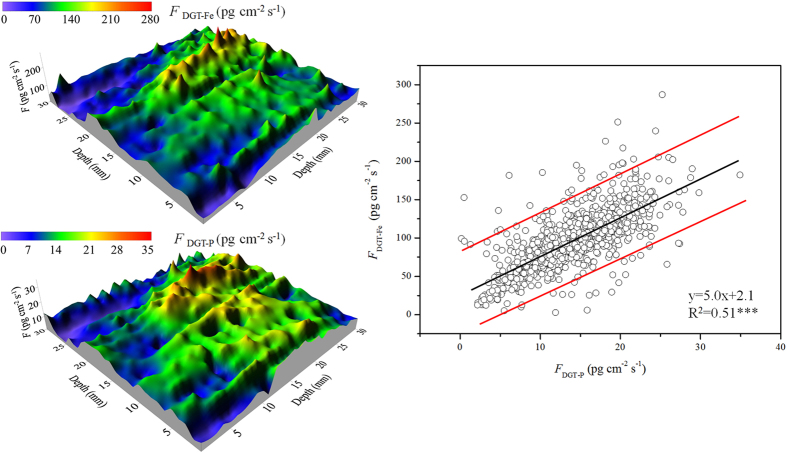
Two-dimensional distributions of DGT-labile Fe and P in the sediment of a site in Lake Taihu and their correlation analyses.

Previous studies have shown distinct vertical and horizontal heterogeneity in the distributions of P, S and trace elements, even on a microscale^38^. For example, a large variation of labile P in Lake Taihu in the horizontal direction has been observed, especially at the sites dominated by macrophytes or close to the lake bank^34^. Verification of the Fe-coupled mobilization of P necessitates an observation of coincident distributions between their DGT-labile species at the 2D level. The labile Fe and P were thus measured at a spatial resolution of 1.0 mm × 1.0 mm using the ZrO-Chelex DGT. Site 8 was selected as a test because it had an intermediate status in terms of the major properties in sediments investigated (e.g., pH value, and Ca, Mn, Fe and P contents) (Tables 1 and 2). Considerable horizontal and vertical heterogeneities appeared for both labile Fe and labile P (Fig. 3). Enriched Fe and P hotspots, were found at depths of approximately 20 mm and widths from 15 to 25 mm. Hotspots with enriched P have been observed in deep layers of sediments in Lake Taihu, which has been attributed to strong decomposition of active organic matter^39^. In this study, labile P and Fe were enriched in a similar zone, reflecting that the enhanced flux of P to the DGT probe is more closely related to Fe redox cycling. Overall, labile Fe and P exhibited a similar change in both the vertical and horizontal directions. A linear correlation was further observed between the *F*_DGT_ of these two species, with most of the data points falling within the 95% confidence interval (Fig. 3). Thus, these results confirmed the Fe-coupled mobilization of P at the 2D level and on a small scale.

According to the principle of ZrO-Chelex DGT measurements mentioned in the Methods, the coincident distributions of DGT-labile Fe and P resulted from a concomitant release of Fe(II) and P from sediment solids, which further demonstrated the mechanism of Fe-coupled mobilization of P in sediments. The value of slope from linear fitting may reflect the ability of reactive Fe (oxyhr)oxides to control P lability in sediments. The low values of slopes (3.1 to 6.3) observed in Sites 1–3 in northern Lake Taihu with pollution character likely demonstrate that there was lower amounts of reactive Fe (oxyhr)oxides to retain P and correspondingly there was a relatively high risk and strength of P release from sediments^34^. In contrast, the high values of slopes (58.3 and 86.1) were observed in Sites 15 and 16 in the submerged macrophytes-dominated region, reflecting a strong control of reactive Fe (oxyhr)oxides on the lability of P in sediments. This is consistent with the report of extremely low concentration of total P in the water column in this region^34^. The slope values at other sites are within the range of the slope values in the above two regions, which is also consistent with their P status in the water column^34^. Furthermore, the dynamics of Fe(II) and P releases remained stable for the 8 sites with a linear fitting, reflecting that the Fe (oxyhr)oxide species retaining the released P prior to release should remain unchanged. This is also true for Sites 1 and 2, which required two linear fittings but had similar slopes. For the remaining 4 sites with large differences in slopes from two linear fittings, the Fe (oxyhydr)oxide species for retaining labile P may be different in the upper and lower sediment layers. The secondary Fe(II) and mixed Fe(II, III) minerals in lower layers likely play a role in regulating the release of P, as mentioned earlier^40^,^41^.

There was no clear trend in the scatter plots for Sites 5 and 12 (Fig. 1). Both sites showed a relatively high *F*_DGT_ of P compared with that of Fe in the upper sediment layer from the SWI to a depth of ~15 mm, which may be attributed to a high-rate of degradation of organic P and polyphosphate, such as pyrophosphate, DNA and phospholipids with short half-lives (3–14 years) in the surface sediments of Lake Taihu^42^. Furthermore, the content of total Ca in the sediment of Site 12 (16.09 g kg^−1^) was far greater than those at the other sites (4.30 to 10.68 g kg^−1^) (Table 1), which may retain labile P at a low level in lower sediment layers through co-precipitation^43^. The content of organic matter in this site was also very high (Table 1), which can reduce the binding capacity of Fe (oxyhydr)oxides for phosphate through competitive sorption or aqueous complexation, and may result in the decoupling of Fe and P^44^.

### Resupply dynamics of Fe and P

The dynamic resupply of sediment solids can be characterized with DGT using *R* if the kinetic exchanges between the solid and pore water are simplified as reversible 1^st^-order desorption-sorption processes^45^. *R* is the ratio of the DGT measured concentration against the pore water concentration (*C*_pw_), reflecting the extent of solid resupply to sustain the *C*_pw_ after DGT uptake.The *R* is calculated as follows:


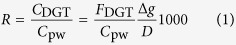


The coincident distributions of P and Fe observed earlier imply that the releases of the two elements should be associated with a coincident resupply of pore water Fe and P from sediment solids during DGT uptake. Accordingly, the *R* from DGT measurement should be correlated between P and Fe^45^.

Considering the above hypothesis, the relationship between the *R* values of P and Fe was investigated in this study. Because it was difficult to determine the *C*_pw_ in the sediments at a high spatial resolution (1 mm in this study), a *C*_DGT_ value was obtained by using a thicker diffusive layer (0.90 mm), which was applied instead of *C*_pw_ to calculate an apparent ratio (*R*′). The use of a thicker diffusive layer, as opposed to the use of a thinner one (0.10 mm), will result in a measured *C*_DGT_ that to a larger extent approaches *C*_pw_ due to a longer time needed for the sediment solids to resupply the depletion of *C*_pw_ during DGT uptake^45^. The ratio of *R*′ using the two diffusive layers can thus assume a role similar to that of *R*, which can be calculated using the *F*_DGT_ derived from eq. 4,


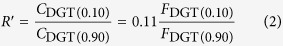


The subscript numbers represent the respective thicknesses of diffusion layers. There should be a correlation for the ratio *F*_DGT(0.10)_/*F*_DGT(0.90)_ between Fe and P if a coincident resupply of pore water Fe and P existed.

**Figure 4:**
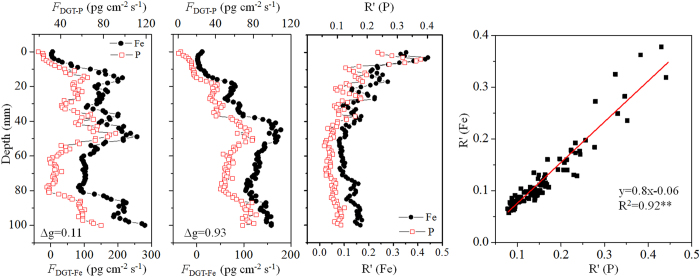
Distributions of labile Fe and P and their corresponding R′ in the sediment of a site in Lake Taihu, measured by a combined ZrO-Chelex DGT probe containing two diffusive layers with 0.10 mm and 0.90 mm thickness, respectively, and a correlation analysis (C) between their *R*′ values.

A combined DGT probe composed of two single DGT probes, containing 0.10 mm and 0.90 mm diffusion layers, was used to obtain the *F*_DGT_ of Fe and P in the sediment of Site 8. In line with the phenomenon observed earlier, a coincident distribution was observed between the *F*_DGT_ of Fe and P measured with DGT containing a 0.10 mm or 0.90 mm diffusion layer (Fig. 4). The calculated *R*′ showed a decreasing trend with depth for both Fe and P, reflecting that the capacity of the sediment solids to sustain the pore water Fe or P became weaker with depth. This phenomenon was likely due to the reductive dissolution of Fe (oxyhydr)oxides from the surface to deep sediments, resulting in a simultaneous decrease in the capacity of solid phase reservoirs to resupply Fe and P to the pore water following removal by the DGT probe. There was a coincident distribution of *R*′ between Fe and P, demonstrated by a positively linear correlation between them. This confirms that there was a coincident resupply of Fe(II) and P from the sediment solids, further supporting the mechanism of Fe-coupled mobilization of P in sediments.

### Identification of Fe (oxyhydr)oxides coupling to P release

This study has provided *in situ* evidence for Fe-coupled mobilization of P, which was demonstrated by a simultaneous release of Fe(II) and P to pore water together with a coincident resupply of pore water Fe(II) and P from sediment solids. As different Fe (oxyhydr)oxides possess a broad degree of reactivity in binding P^37^, it is vital to identify which Fe (oxyhydr)oxides retained the labile P fraction prior to its release from DGT perturbance. An earlier investigation indicated that the speciation of P in sediments was likely regulated by the Fe-ox1 and Fe-ox2 (Supplementary Figs S1–S4), and thus the relationship between DGT-labile Fe/P and the two extracted Fe/P fractions was investigated. The investigation was confined in the surface 30 mm layer, as P was found to be highly labile in this layer and may exert a dominant effect on P release to the overlying water^34^. Moreover, the Fe/P ratio is used for the investigation because it is far more stable than the respective DGT mass or extracted concentrations of Fe and P.

**Figure 5:**
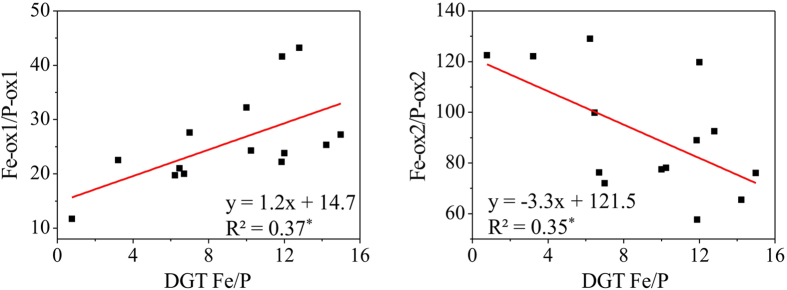
Correlation analysis of DGT Fe/P with easily reducible or reducible Fe/P.

The results showed a significantly positive correlation for the Fe/P ratio between the DGT labile and easily reducible fractions, whereas a negative correlation was found between the DGT labile and reducible fractions (two sites were excluded from the investigation because of their extremely high DGT Fe/P values) (Fig. 5). This demonstrates that Fe-ox1, mainly ferrihydrite, should act as a binding phase for labile P prior to its release. This is likely because they are highly sensitive to redox conditions, causing a rapid dissolution and coupled release of P when the redox condition in sediment changes from oxic to anoxic. As the DGT perturbance reflects a temporary release process of P in sediments, it simultaneously demonstrates that the P-ox1 may be responsible for a short-term, high-magnitude release of sediment P to the overlying water after the onset of anoxia, while the P-ox2 may act as a less labile P fraction which sustains P release which is predicted to coincide with a decrease in the overall flux of P. As a result, the two P fractions may be a major source of P to the water column during warm seasons^15^,^27^.

## Methods

### Description of the sampling sites

**Figure 6:**
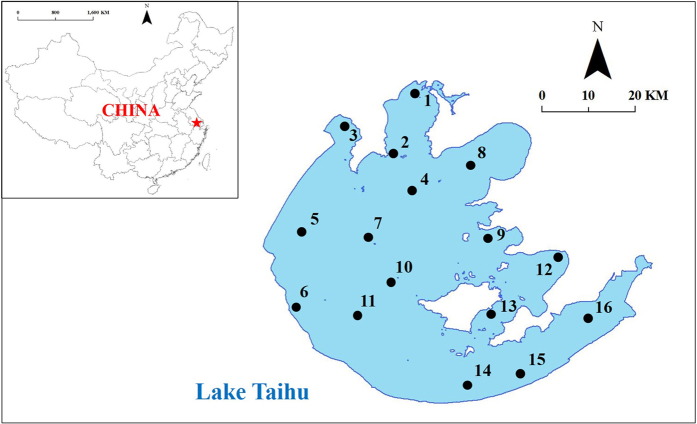
The distribution of sampling sites in Lake Taihu. The map is Reprinted (adapted) with permission from^46^. Copyright (2009) American Chemical Society.

Taihu is the 3^rd^ largest lake in China. It is a typical eutrophic lake, with the water trophic level declining from the North and Northwest to South and Southeast^46^. A total of 16 sites were selected as representative of different ecotypes in Lake Taihu (Fig. 6). Sites 1 to 5 were located in the north and northwest regions, which are frequently dominated by algae. Sites 12 to 16 were located in the southeastern bays, which are dominated by macrophytes. The other sites had no visible macrophyte coverage, but occasionally suffered from algal blooms. The positions and ecological statuses of the sites are shown in Supplementary Table S1.

### Preparation and deployment of DGT probes

Principle of ZrO-Chelex DGT measurements is demonstrated in Supplementary Information and Fig. S5. Previous studies have shown that the uses of thin diffusion layers down to 0.01 mm thickness do not differ in terms of DGT response from that of a conventional diffusion layer with a typical thickness of 0.93 mm^47^,^48^. A modification of the ZrO-Chelex DGT was thus performed by removing the diffusive gel and using a Durapore^®^ PVDF membrane (HVLP00010, Millipore) as the diffusion layer. The hydrophilic membrane has a pore size of 0.45 μm and a thickness of 0.10 mm. This modification can obtain a rapid DGT uptake of Fe and P and shorten the deployment time to 1 d. The use of a thin diffusion layer should also result in stronger releases of Fe and P in sediments from DGT perturbance, rendering this technique more sensitive in reflecting their possible coincident behaviors. The blanks of the ZrO-Chelex DGT for Fe and P were detected using 6 gel discs with a diameter of 2.5 cm and the values are 0.048 μg cm^−2^ and 0.006 μg cm^−2^, respectively. The limits of detection (calculated as 3 times the standard deviation of the blanks) for Fe and P were 0.028 μg cm^−2^ and 0.011 μg cm^−2^, respectively.

The ZrO-Chelex binding gel was provided by Easysensor Ltd. (www. easysensor. net), which was prepared according to Xu *et al*.^32^. The gel (with the ZrO-Chelex settled surface upward) was covered with the PVDF membrane. The gels and PVDF membrane were sealed in a holder made by Perspex, with a sheet of sponge attached on the back of the holder to mark the sediment-water interface (SWI) after retrieving the probe from the sediments^34^. At Site 8, this type of DGT probe was bound back-to-back with a DGT probe assemblied by an agarose cross-linked polyacrylamide (APA) diffusive gel with a thickness of 0.80 mm^49^ to investigate the resupply dynamics of Fe and P in sediments. All the DGT probes were manufactured in a class 1000 cleanroom.

The ZrO-Chelex DGT probes were deoxygenated with nitrogen overnight and stored in a container filled with deoxygenated 0.01 M NaCl (Sinopharm Chemical Reagent Co., Ltd., SCRC, Beijing; AR grade) prepared using deionized water (Millipore, >18 MΩ cm). The probes were transported to the sampling sites and inserted into the sediments using a releasing device^39^ during October, 2014 and were deployed for 24 h. After retrieval, each probe was rinsed rapidly using lake water to remove visible sediment particles that were attached, followed by using deionized water for a further clean. They were placed in a container sealed at air temperature to prevent moisture loss, and were transported to the laboratory. The containers used for storing the DGT probes have been washed using 10% HNO_3_.

### Sampling of sediments

The sediment cores (~10 cm) at each site were collected using a gravity core sampler during the deployment of DGT. Each core was sliced into 0.5 cm sections down to 5 cm, and then into 1.0 cm sections down to 10 cm under a N_2_ atmosphere. The sediment samples were lyophilized at −80 °C, sieved to pass through a 100-mesh sieve and then stored at 4 °C until analysis.

### Sample analyses

The ZrO-Chelex binding gels were removed from the DGT probes and sliced into 1 mm sections along the vertical direction using a multi-bladed ceramic cutter. For Site 8, the gel was also sliced into a square array using a previously reported method^50^, and each gel square had a size of 1.0 mm × 1.0 mm. Each slice or gel square was transported into a microwell placed in a 96- or 384-microwell plate holder. Fe and P bound in the gel were progressively eluted using 1.0 M HNO_3_ (SCRC, AR grade) and 1.0 M NaOH (SCRC, AR grade) according to Xu *et al*.^32^. The concentrations of Fe and P in the extracts were detected using the molybdenum blue and phenanthroline colorimetric methods, respectively, using an Epoch Microplate Spectrophotometer (BioTek, USA)^17^.

Basic chemical properties of the sediment samples were analyzed using standard methods^51^, with three replicates performed for each parameter. The organic matter content in the sediments was measured by TOC using a TOC analyzer (TOC-V CPN, Shimadzu). The pH was analyzed in a 1:10 solid:liquid ratio suspension using a pH electrode (PB-10, Sartorius). The pseudo total concentrations of P, Fe, Al, Mn and Ca in the sediments were measured using an ICP-AES (Profile D, Leeman) after fusing 0.05 g (DW) of sediment with 0.2 g of LiBO_2_, followed by dissolution with 4% HNO_3_^52^. The accuracy of the analysis was checked using standard reference material for lake sediments (GBW07436, Center for Standard Reference of China). Easily reducible (Fe-ox1, ferrihydrite and lepidicrocite) and reducible (Fe-ox2, goethite, hematite and akaganéite) Fe (oxyhydr)oxides in sediments were obtained through sequential extractions of the sediments with hydroxylamine-HCl solution for 48 h and dithionite for 2 h after removal of carbonate Fe with Na acetate solution for 24 h^36^. The concentrations of Fe and P in the elution solutions were measured using the ICP-AES. Total P, total dissolved P (TDP) and dissolved reactive P (DRP) in water samples were measured using the molybdenum blue method following standardized treatments^51^.

### Calculation

The DGT measurement is generally interpreted as the time-averaged concentration at the diffusion layer-sediment interface (*C*_DGT_)^29^,


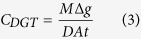


where Δ*g* (cm) is the thickness of the diffusion layer, *D* (cm^2^ s^−1^) is the diffusion coefficient of the analyte in the diffusion layer, *t* (s) is the deployment time, *A* (cm^2^) is the exposure area of the gel, and *M* (μg) is the corresponding accumulated mass over the deployment time.

When a thin diffusion layer is used, the measured *C*_DGT_ is far lower than the pore water concentration. To avoid the improper interpretation of the DGT measured result as a pore water concentration^53^, the DGT-labile Fe or P was interpreted herein as the flux (ng cm^−2^ s^−1^), as used by others^47^,^53^,^54^,^55^:


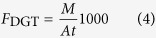


### Correlation analysis

The correlations between different couples of Fe and P species, including Fe-ox1 and P-ox1, Fe-ox2 and P-ox2, and DGT-labile Fe and P, were analyzed using linear fitting with the the least squares approach. The fitting between Fe and P from chemical fractionation was performed using the data from the SWI to a depth at which the subsequent data (just below the depth) evidently deviated from the data group included. Generally the data at the entire depths could be used in fitting a line in the major sites, with the significant levels for all the correlation coefficients (R) *p* < 0.05. The fitting between Fe and P from DGT measurement was generally performed using the data at the entire depths if the significant levels reached *p* < 0.001. In another case, the fitting was performed using the data from the SWI to a depth at which the subsequent data (just below the depth) evidently deviated from the data group included. The remaining data was then used for fitting another line. Both the significant levels from two line fittings also reached *p* < 0.001.

## Additional Information

**How to cite this article**: Ding, S. *et al*. In situ, high-resolution evidence for iron-coupled mobilization of phosphorus in sediments. *Sci. Rep*. **6**, 24341; doi: 10.1038/srep24341 (2016).

## Supplementary Material

Supplementary Information

## Figures and Tables

**Table 1:** Basic properties of the sediments.

NO.	pH	TOC	Total Ca	Total Mg	Total Mn	Total Al
		(mg g^−1^)	(mg g^−1^)	(mg g^−1^)	(mg g^−1^)	(mg g^−1^)
1	6.82	9.89	7.45	5.24	0.63	59.79
2	6.81	8.26	4.92	5.23	0.68	59.60
3	6.69	16.14	7.09	5.59	1.2 8	60.56
4	7.03	7.12	4.30	3.71	0.44	53.25
5	6.87	9.89	7.98	4.82	0.78	54.60
6	7.18	8.86	6.30	5.65	0.52	58.30
7	7.01	5.89	6.67	5.34	1.07	53.20
8	7.19	4.59	10.68	7.07	0.62	68.46
9	7.01	8.60	8.76	5.67	0.49	58.54
10	6.93	8.23	5.78	5.95	0.69	50.66
11	7.46	7.18	9.80	5.26	0.73	53.64
12	6.99	19.47	16.09	3.51	0.37	51.62
13	6.59	12.83	9.28	4.25	0.55	52.96
14	7.24	9.26	9.61	7.81	1.10	70.27
15	6.78	11.89	8.82	4.90	0.51	49.46
16	6.81	23.37	8.63	3.92	0.44	52.97
Ave.	6.96	10.72	8.26	5.25	0.68	56.74
Min.	6.59	4.59	4.30	3.51	0.37	49.46
Max.	7.46	23.37	16.09	7.81	1.28	70.27

**Table 2:** Summary for Fe and P contents (g kg^−1^), proportions (%, for easily reducible and reducible species), and concentration ratios of Fe to P (Fe/P) in sediments of Lake Taihu.

NO.	Fe					P							
	Total	Easily reducible		Reducible		Total	Easily reducible		Reducible		Fe/P		
	Content	Content	Proportion	Content	Proportion	Content	Content	Proportion	Content	Proportion				Total	Easily reducible	Reducible
1	34.92	5.60	16.05	8.02	22.97	0.50	0.27	53.33	0.08	16.08	69.8	21.0	99.8
2	28.96	5.62	19.39	7.12	24.59	0.46	0.25	54.35	0.06	12.68	63.0	22.5	122.1
3	31.10	7.28	23.40	9.19	29.55	0.95	0.62	65.22	0.08	7.89	32.7	11.7	122.5
4	28.77	3.62	12.59	6.88	23.91	0.36	0.16	45.24	0.08	21.48	79.9	22.2	89.0
5	28.34	6.58	23.21	7.53	26.55	0.54	0.33	61.73	0.06	10.80	52.5	19.7	129.0
6	32.28	6.90	21.38	7.10	22.00	0.55	0.27	49.61	0.11	19.70	58.7	25.3	65.5
7	33.92	5.29	15.60	6.54	19.29	0.83	0.38	45.18	0.08	9.04	40.9	14.1	87.3
8	37.19	7.81	21.00	3.85	10.35	0.49	0.28	57.82	0.05	10.91	75.9	27.6	72.0
9	32.15	4.76	14.81	7.60	23.63	0.46	0.18	38.04	0.10	21.74	69.9	27.2	76.0
10	29.65	5.42	18.27	9.37	31.60	0.57	0.22	39.07	0.12	21.05	52.0	24.3	78.1
11	25.51	4.78	18.75	7.45	29.19	0.47	0.20	42.80	0.06	13.23	54.3	23.8	119.8
12	25.57	4.85	18.97	7.20	28.16	0.45	0.12	25.93	0.13	27.78	56.8	41.6	57.6
13	26.42	5.89	22.28	6.83	25.87	0.43	0.18	42.55	0.09	20.50	61.4	32.2	77.5
14	45.44	10.07	22.17	4.71	10.37	0.49	0.23	47.62	0.05	10.39	92.7	43.2	92.5
15	25.14	3.61	14.36	5.33	21.21	0.48	0.18	37.58	0.07	14.59	52.4	20.0	76.2
16	25.44	4.30	16.91	3.71	14.58	0.57	0.20	35.09	0.06	10.23	44.6	21.5	63.6
Ave.	30.68	5.77	18.70	6.78	22.74	0.54	0.25	46.32	0.08	15.51	59.9	24.9	89.3
Min.	25.14	3.61	14.36	3.71	10.35	0.36	0.12	25.93	0.05	7.89	32.7	11.7	63.6
Max.	45.44	10.07	23.4	9.37	31.6	0.95	0.62	65.22	0.12	27.78	92.7	43.2	129.0
