# Supplementary material for: In situ, high-resolution evidence for iron-coupled mobilization of phosphorus in sediments
Source: Sci Rep. 2016 Apr 18;6:24341. doi: 10.1038/srep24341 (PMC4834547; doi:10.1038/srep24341)
Supplement: Supplementary Information [file srep24341-s1.doc]

**Supplementary Material**

**In situ, high-resolution evidence for iron-coupled mobilization of phosphorus in sediments**

Shiming Ding^1,*^, Yan Wang^1^, Dan Wang^1^, YangYang Li^1,2^, Mengdan Gong^1^, Chaosheng Zhang^3^

^1^State Key Laboratory of Lake Science and Environment, Nanjing Institute of Geography and Limnology, Chinese Academy of Sciences, Nanjing 210008, China

^2^School of Chemical Engineering, Nanjing University of Science and Technilogy, Nanjing 210094, China

^3^GIS Centre, Ryan Institute and School of Geography and Archaeology, National University of Ireland, Galway, Ireland

^*^[smding@niglas.ac.cn](mailto:smding@niglas.ac.cn)

**Contents**

**Text** Principle of ZrO-Chelex DGT measurements

**Table S1** The GPS positions and dominant ecological status of the sampling sites in Lake Taihu.

**Fig. S1** The vertical distributions of easily reducible Fe and associated P in sediments of Lake Taihu.

**Fig. S2** The vertical distributions of reducible Fe and associated P in sediments of Lake Taihu.

**Fig. S3** Correlation analysis between easily reducible Fe and associated P.

**Fig. S4** Correlation analysis between reducible Fe and associated P.

**Fig. S5** Schematic diagram of Fe-coupled release of P in sediments and its detection by ZrO-Chelex DGT.

**Principle of ZrO-Chelex DGT measurements.**

Details of the ZrO-Chelex DGT theory have been reported previously^1^. This DGT was developed based on a mixed binding gel (ZrO-Chelex gel) impregnated with amorphous zirconium hydroxide (Zr-oxide) and Chelex-100 resin for binding dissolved P (orthophosphate) and Fe(II) ions, respectively. The binding gel was usually overlain by a diffusion layer composed of a polyacrylamide hydrogel and a membrane filter, in which both Fe and P ions diffuse at known diffusion rates. When a ZrO-Chelex DGT probe was deployed in sediments, soluble Fe(II) and P in pore water diffuse into the probe through the diffusion layer and are immediately bound in the binding gel. Linear concentration gradients of Fe and P will form in the diffusive layer within a few minutes, whereas the effective concentrations of Fe and P at the binding gel-diffusion gel interface remain zero during the deployment.

Once a ZrO-Chelex DGT probe is deployed in sediments, an instantaneous depletion of pore water Fe and P occurs at the surface of the DGT probe, leading to releases of Fe and P from sediment solids to resupply their pore water concentrations. As a consequence, the DGT-labile Fe and P mostly represent local releases from their labile pools, which are weakly bound onto the sediment solids when the deployment time is relatively long (e.g., one to several days). According to the mechanism of Fe-coupled mobilization of P, the reductive dissolution of Fe (oxyhy)droxides will lead to a concomitant release of P to the pore water (Fig. S5). This will result in a coincident distribution between DGT-measured Fe and P, provided that the ability of Fe (oxyhy)droxides in to regulate the release of P remained stable in the sediments. Recent studies suggested that a portion of the produced Fe(II) can form secondary Fe(II) and mixed Fe(II, III) minerals, such as magnetite, siderite, and green rust prior to its release to solution or prior to the formation of more stable minerals (e.g., vivianite)^2-4^. These intermediate products may act as temporary carrier phases in retaining P^5, 6^. As a consequence, *in situ* observation of a coincident distribution of DGT-measured Fe and P resulted from a concomitant release of Fe and P, whether from the dissolution of Fe(III) oxyhydroxides or secondary Fe(II) and mixed Fe(II, III) minerals, can provide *in situ* evidence for the Fe-coupled mobilization of P. Such a method with DGT has been successfully employed to reveal the couplings between Mn and Co, Fe and As, and sulfide and trace metals in sediments^7-9^.

**Table S1** The GPS positions and dominant ecological status of the sampling sites in Lake Taihu

| NO. | GPS position (in degrees) | | Ecological status | |
| --- | --- | --- | --- | --- |
|  | Latitude | Longitude |  |  |
| 1 | 31.51 | 120.19 | Frequently dominated by algae, and having visible macrophyte coverage | |
| 2 | 31.41 | 120.15 |  |  |
| 3 | 31.45 | 120.06 |  |  |
| 4 | 31.34 | 120.18 |  |  |
| 5 | 31.26 | 120.98 |  |  |
| 6 | 31.11 | 120.97 | Occasionally suffered from algal blooms, and no visible macrophyte coverage | |
| 7 | 31.25 | 120.10 |  |  |
| 8 | 31.39 | 120.30 |  |  |
| 9 | 31.24 | 120.33 |  |  |
| 10 | 31.16 | 120.14 |  |  |
| 11 | 31.09 | 120.08 |  |  |
| 12 | 31.21 | 120.46 | Covered by macrophytes | *P.malaianus, N.peltatum* |
| 13 | 31.10 | 120.34 |  | *P.malaianus, N.peltatum, V.natans, H.verticillata* |
| 14 | 31.97 | 120.30 |  | *P.malaianus, N.indica* |
| 15 | 31.98 | 120.38 |  | *P.macckianus, P.malaianus, C.demersum, V.natans, H.verticillata, T.quadrispinosa, N.peltatum, N.indica* |
| 16 | 31.09 | 120.51 |  | *P.macckianus, C.demersum, V.natans, H.verticillata* |

**Fig. S1** The vertical distributions of easily reducible Fe and associated P in sediments of Lake Taihu. The depth at zero is the sediment-water interface.

**Fig. S2** The vertical distributions of reducible Fe and associated P in sediments of Lake Taihu. The depth at zero is the sediment-water interface.

**Fig. S3** Correlation analysis between easily reducible Fe and associated P. The numbers in parentheses show the depth for fitting. The one and two asterisks indicate significance level at *p*<0.05 and *p*<0.01, respectively.

**Fig. S4** Correlation analysis between reducible Fe and associated P. The numbers in parentheses show the depth for fitting. The one and two asterisks show significance level at *p*<0.05 and *p*<0.01, respectively.

**Fig. S5** Schematic diagram of Fe-coupled release of P in sediments and its detection by ZrO-Chelex DGT.

**References**

1. Xu, D. et al. Diffusive Gradients in Thin Films Technique Equipped with a Mixed Binding Gel for Simultaneous Measurements of Dissolved Reactive Phosphorus and Dissolved Iron. *Environ. Sci. Technol.* **47**, 10477-10484 (2013).

2. Lovley, D.R. Microbial Fe(III) reduction in subsurface environments. *FEMS Microbiol. Rev.* **20**, 305-313 (1997).

3. Ruby, C. et al. Oxidation modes and thermodynamics of FeII–III oxyhydroxycarbonate green rust: Dissolution–precipitation versus in situ deprotonation. *Geochim. Cosmochim. Acta* **74**, 953-966 (2010).

4. Slomp, C.P. et al. Coupled Dynamics of Iron and Phosphorus in Sediments of an Oligotrophic Coastal Basin and the Impact of Anaerobic Oxidation of Methane. *PLoS ONE* **8**, e62386 (2013).

5. Benali, O., Abdelmoula, M., Refait, P. & Génin, J.-M.R. Effect of orthophosphate on the oxidation products of Fe(II)-Fe(III) hydroxycarbonate: the transformation of green rust to ferrihydrite. *Geochim. Cosmochim. Acta* **65**, 1715-1726 (2001).

6. Génin, J.-M.R., Refait, P., Bourrié, G., Abdelmoula, M. & Trolard, F. Structure and stability of the Fe(II)–Fe(III) green rust “fougerite” mineral and its potential for reducing pollutants in soil solutions. *Appl. Geochem.* **16**, 559-570 (2001).

7. Bennett, W.W. et al. Investigating Arsenic Speciation and Mobilization in Sediments with DGT and DET: A Mesocosm Evaluation of Oxic-Anoxic Transitions. *Environ. Sci. Technol.* **46**, 3981-3989 (2012).

8. Gao, Y., van de Velde, S., Williams, P.N., Baeyens, W. & Zhang, H. Two-dimensional images of dissolved sulfide and metals in anoxic sediments by a novel diffusive gradients in thin film probe and optical scanning techniques. *TrAC Trends Anal. Chem.* **66**, 63-71 (2015).

9. Zhang, H. et al. (important)Localised remobilization of metals in a marine sediment. *Sci. .Total Environ.* **296**, 175-187 (2002).
